# Laryngotomy as a Preliminary to Mouth and Throat Operations

**Published:** 1903-01-17

**Authors:** 


### LARYNGOTOMY AS A PRELIMINARY TO MOUTH AND THROAT OPERATIONS.

IN all large operations about the mouth and throat four special dangers have to be guarded against, namely, sepsis, septic affections of the lungs, special risks connected with anæsthesia, and hæmorrhage. Speaking as to the best means of averting these dangers Mr. Butlin[1] recommends the performance of a preliminary laryngotomy. It is not unnatural, he says, that the question of a preliminary tracheotomy should have presented itself to those engaged in operations on the mouth and throat, but it has never been generally popular among surgeons. The operation itself is tedious; there is frequently more loss of blood than is desirable; the wound may be as troublesome to deal with as that of the principal operation; and there may for several days be difficulty in coughing up discharges which collect at the back of the throat. Yet, in spite of its disadvantages, tracheotomy with the use of Hahn's tube is a wonderful preliminary to operations in or immediately above the larynx. For operations higher up, however, such as the removal of the whole or a part of the tongue, or for operations in the tonsillar region, the palate, naso-pharynx, or the inside of the cheeks, the best preliminary operation is a laryngotomy. The best instruments for the purpose are a scalpel, a strong-pointed curved needle on a handle, a straight sharp-pointed steel director, a blunt-pointed director, and a tracheotomy dilator, the ends of which should be smooth and not bulbous. The tube should have a collar like that of a tracheotomy tube and should be introduced on a solid guide which projects well beyond the tube and terminates almost in a point. In performing the operation a transverse incision about two-thirds of an inch long is made over the crico-thyroid membrane and into, but not deeply into, the subcutaneous tissue, just exposing, but not wounding, the subcutaneous vessels. The curved needle is then thrust carefully through the membrane as near the middle as possible, and between the veins if they are seen. On this as a guide the straight sharp-pointed director is thrust through the membrane, and, the needle being withdrawn, the blunt director is run through into the larynx along the groove of the sharp one. The two directors are then forcibly separated, so as to allow of the introduction of the dilator, when they are withdrawn, and the opening being then dilated, the tube and its guide are introduced either before or after the dilator is withdrawn. Thus performed the operation rarely occupies more than a minute in the doing. There is usually no bleeding, and the tube is withdrawn as soon as the chief operation is completed. The wound left hardly requires any dressing and soon heals. If by accident hæmorrhage should occur and is abundant the wound must be opened a little wider and the vessels clamped or tied. A bulbous dilator must not be used, for the space left at the side of the tube is so small that there may be difficulty in getting the dilator out again. When the preliminary operation has been completed a sponge fastened to a stout string or piece of tape is placed at the back of the throat, being thrust well down so as to fill the lower pharynx and cover the larynx, thus preventing the passage of discharges in that direction. The object of having a collar on the tube is to prevent the lower end of the tube tilting up, and thus causing interference with the respiration, as it otherwise might do, when the sponge is placed in the back of the throat. If this accident should nevertheless occur, a pair of clamp forceps fixed to the collar will serve to hold the tube in its place. This preliminary laryngotomy not only diminishes the danger of the chief operation but it enables the surgeon to go about his work deliberately and with almost the same facility as if the disease were in an open part of the body, which is a most important advantage.

[1] The Clinical Journal, Dec. 10.

